# Successful management of presumed *Candida* endogenous endophthalmitis with oral voriconazole

**DOI:** 10.4103/0301-4738.53058

**Published:** 2009

**Authors:** Raju Biju, Daniel Sushil, Nainan K Georgy

**Affiliations:** Ranjini Eye Care, Cochin, India; 1 Grace Eye Hospital, Cochin, India; 2 Lakeshore Hospital and Research Center, Cochin, India

**Keywords:** *Candida* endophthalmitis, endophthalmitis, endogenous endophthalmitis, fungal endophthalmitis, voriconazole

## Abstract

Endogenous fungal endophthalmitis is most commonly caused by *Candida* species and usually occurs in patients with chronic diseases such as diabetes mellitus and renal insufficiency. Voriconazole, a broad-spectrum triazole antifungal agent, attains therapeutically significant concentrations in the vitreous cavity after systemic administration. We report, the successful management of presumed endogenous *Candida* endophthalmitis in a patient with multiple diseases and unstable systemic status with oral voriconazole. Though fungal endophthalmitis has been successfully treated with a combination of intravenous and intravitreal voriconazole, to the best of our knowledge this is the first report in ophthalmic literature (Medline Search) on the treatment of fungal endophthalmitis with only the oral route of administration of voriconazole.

Patients with chronic diseases such as diabetes mellitus and renal insufficiency are at the risk of developing endogenous fungal endophthalmitis.[1] Intravenous and intravitreal voriconazole, a new triazole antifungal agent has been used to treat fungal endophthalmitis.[2] We report a case of presumed endogenous endophthalmitis in a patient who was on regular hemodialysis for renal insufficiency and compromised systemic status treated successfully only with oral voriconazole. To the best of our knowledge this is the first report in ophthalmic literature (Medline Search) on the treatment of fungal endophthalmitis with only the oral route of administration of voriconazole.

## Case Report

A 73-year-old male, diabetic for 11 years, hypertensive for the past five years and with ischemic heart disease presented with gradually progressive painless loss of vision in the right eye of three weeks duration. He had an indwelling double lumen subclavian catheter and was undergoing dialysis once every three days for chronic renal failure. The patient was on oral prednisolone 10 mg daily for systemic myasthenia gravis since 1981. He had undergone cataract extraction with intraocular lens (IOL) implantation in the right eye three years ago.

**Figure 1:**
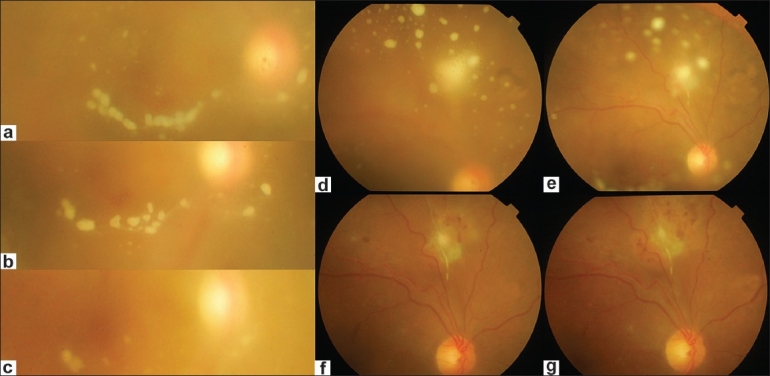
Regression of vitreous exudates following treatment with voriconazole (a) String of beads at first visit; (b) One week after starting voriconazole – note the spreading away of the “beads”; (c) At four weeks follow-up; (d) At first visit: note the extensive vitreous exudation and fuzzy margins of the chorioretinitis patch; (e) The margins of the lesion become discrete along with reduction in vitreous exudation one week after voriconazole. Note the focus of arteritis close to the lesion; (f) Lesion shows signs of scaring at eight weeks; (g) Complete resolution at final visit

His visual acuity was 20/100, N36 and 20/20, N6 in the right and left eye respectively. The anterior segment evaluation of the right eye showed 2+ cells and a sluggish pupil. The right eye was pseudophakic with a clear posterior capsule. The fundus evaluation showed multiple cotton-ball opacities in the vitreous and few had coalesced to a ‘string of beads” appearance [Fig. 1]. An area of active chorioretinitis and arteritis along the superotemporal arcade was noted. Slit-lamp biomicroscopy showed numerous vitreous cells. The clinical picture was typical of endogenous endophthalmitis in the right eye most probably due to *Candida species*.[1] Fundus evaluation of the left eye showed an old branch retinal vein occlusion with photocoagulation scars and changes suggestive of mild non-proliferative diabetic retinopathy.

Pars plana vitrectomy and intravitreal amphotericin-B was planned at first visit itself. But as he was due for dialysis the same day, after discussing with the nephrologists, taking into account the multiple co-morbid conditions of the patient he was started on voriconazole. Oral voriconazole 200 mg twice daily was started along with prednisolone acetate eye drops every 2 h and atropine sulphate 1% eye drops thrice daily. When he came for follow-up two days after dialysis, his general condition as well as the ocular status was much better. The cellular reaction had reduced with 1+ cells and the media clarity was marginally better. Therefore, we thought it was unethical for the sake of a microbiologic diagnosis, to subject him to an invasive procedure. Retrospectively we believe that the source of the infection was the indwelling catheter. Oral prednisolone (10 mg daily) was continued along with voriconazole.

**Figure 2:**
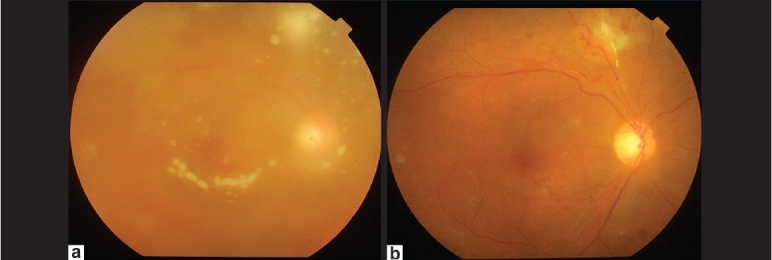
(a) Fundus picture at first visit showing extensive vitreous exudation and string of beads appearance along with an active area of chorioretinitis; (b) Fundus picture at final follow-up shows a normal posterior pole with minimal vitreoretinal traction at the site of resolved chorioretinitis

Within a week of starting oral voriconazole, his visual acuity improved to 20/80 and the media started clearing. The string of beads had started to break away and reduce in size. The fuzzy margins of the cotton-ball opacities and the chorioretinitis patch were becoming discrete. Oral voriconazole was continued for three weeks. After three weeks, the media cleared completely. Voriconazole and the topical medications were stopped. He was evaluated six weeks after stopping voriconazole. His visual acuity improved to 20/20 and N6 in the right eye. The string of pearls had completely disappeared and the chorioretinitis patch had healed [Fig. 2].

## Discussion

Voriconazole, a triazole antifungal agent, has a broad spectrum of action against *Aspergillus* species, *Candida* species and other fungi. It has 96% bioavailability after oral administration and achieves therapeutic aqueous and vitreous levels in non-inflammed human eyes.[2] Because of these favorable properties and good tolerability, voriconazole is a better drug to combat fungal endophthalmitis when compared to amphotericin B. In this patient, endogenous endophthalmitis due to presumed *Candida* species was treated successfully with oral voriconazole alone. Because of his multiple co-morbid conditions a trial of voriconazole by oral route was preferred over the conventional management of vitrectomy and intravitreal injection.

Long-term use of conticosteroids or immunosuppressive agents, and use of indwelling intravenous catheters are risk factors for endogenous endophthalmitis, the infection reaching the eye by hematogenous spread.[1,3] Chronic diseases such as diabetes mellitus and renal insufficiency also increase the risk. In this case, the patient, a chronic diabetic, had been on long-term dialysis and was on oral prednisolone.

*Candida* endophthalmitis classically shows a patch of chorioretinitis with multiple cotton-ball opacities in the vitreous cavity which may join together to give the “string of pearls” appearance.[1] Though microbiologic evidence of the organism causing endophthalmitis is lacking in this case, the typical presentation and the response to therapy with voriconazole, give compelling evidence to an endogenous fungal endophthalmitis due to *Candida* species.

Intravenous and intravitreal voriconazole has been used to treat drug-resistant fungal endophthalmitits.[4] The usual practice is to combine the two routes to achieve a therapeutic concentration in the eye. In a series of five patients of culture-proven *Candida* endogenous endophthalmitis, a combination therapy of intravenous as well as oral voriconazole along with capsofungin, the first approved agent from a new class of antifungals, the echinocandins, resulted in favorable outcomes. [5]

The percentages of voriconazole concentration achieved in the vitreous and aqueous in non-inflammed eyes after two doses of 400 mg orally were 38.1% and 53.0%, respectively[2] and this case illustrates that selected patients of endogenous *Candida* endophthalmitis can be successfully treated with only oral administration of voriconazole.
